# Tracer kinetic modelling for DCE-MRI quantification of subtle blood–brain barrier permeability

**DOI:** 10.1016/j.neuroimage.2015.10.018

**Published:** 2016-01-15

**Authors:** Anna K. Heye, Michael J. Thrippleton, Paul A. Armitage, Maria del C. Valdés Hernández, Stephen D. Makin, Andreas Glatz, Eleni Sakka, Joanna M. Wardlaw

**Affiliations:** a Neuroimaging Sciences, University of Edinburgh, Chancellors Building, 49 Little France Crescent, Edinburgh EH16 4SB, UK; b Department of Cardiovascular Science, University of Sheffield, Medical School, Beech Hill Road, Sheffield S10 2RX UK

**Keywords:** Blood–brain barrier, Dynamic contrast-enhanced MRI, Tracer kinetic modelling, Cerebral small vessel disease

## Abstract

There is evidence that subtle breakdown of the blood–brain barrier (BBB) is a pathophysiological component of several diseases, including cerebral small vessel disease and some dementias. Dynamic contrast-enhanced MRI (DCE-MRI) combined with tracer kinetic modelling is widely used for assessing permeability and perfusion in brain tumours and body tissues where contrast agents readily accumulate in the extracellular space. However, in diseases where leakage is subtle, the optimal approach for measuring BBB integrity is likely to differ since the magnitude and rate of enhancement caused by leakage are extremely low; several methods have been reported in the literature, yielding a wide range of parameters even in healthy subjects. We hypothesised that the Patlak model is a suitable approach for measuring low-level BBB permeability with low temporal resolution and high spatial resolution and brain coverage, and that normal levels of scanner instability would influence permeability measurements. DCE-MRI was performed in a cohort of mild stroke patients (*n* = 201) with a range of cerebral small vessel disease severity. We fitted these data to a set of nested tracer kinetic models, ranking their performance according to the Akaike information criterion. To assess the influence of scanner drift, we scanned 15 healthy volunteers that underwent a “sham” DCE-MRI procedure without administration of contrast agent. Numerical simulations were performed to investigate model validity and the effect of scanner drift. The Patlak model was found to be most appropriate for fitting low-permeability data, and the simulations showed *v*_p_ and *K*^Trans^ estimates to be reasonably robust to the model assumptions. However, signal drift (measured at approximately 0.1% per minute and comparable to literature reports in other settings) led to systematic errors in calculated tracer kinetic parameters, particularly at low permeabilities. Our findings justify the growing use of the Patlak model in low-permeability states, which has the potential to provide valuable information regarding BBB integrity in a range of diseases. However, absolute values of the resulting tracer kinetic parameters should be interpreted with extreme caution, and the size and influence of signal drift should be measured where possible.

## Highlights

• We performed DCE-MRI in 201 patients with a range of small vessel disease severity.
• We tested tracer kinetic model performance via simulations and statistical analysis.
• The Patlak model was optimal for assessing leakage in normal tissues and lesions.
• Scanner drift leads to substantial errors in measured tracer kinetic parameters.
• DCE-MRI measurements of subtle leakage should be interpreted with caution.

## Introduction

Imaging and biochemical investigations suggest that breakdown of the blood–brain barrier (BBB) may be implicated in ageing and the pathophysiology of several diseases, including cerebral small vessel disease, lacunar stroke, vascular dementia and Alzheimer's disease (Farrall and Wardlaw, 2009, Iadecola, 2013, Wardlaw et al., 2013a). Reliable in vivo methods for measuring microvascular integrity are therefore essential to investigate the mechanisms underlying such diseases and to assess potential therapies. The cerebrospinal fluid (CSF) to plasma albumin ratio is a widely accepted in vivo marker of BBB integrity but is non-specific and highly invasive.

The most widely used imaging method for assessing BBB integrity and other microvascular properties is dynamic contrast-enhanced MRI (DCE-MRI), in which a paramagnetic contrast agent is injected and the time dependence of the resulting *T*_1_-weighted image enhancement measured. There are many approaches to analysing such data, from simple heuristic measures to a growing library of tracer kinetic models (Sourbron and Buckley, 2012), which aim to estimate physiological parameters including blood flow, blood volume and the rate of extravascular leakage. These techniques have been developed and applied primarily in neurooncology and body imaging, where contrast uptake in tissue is typically large and rapid. However, the optimal modelling approach for research in diseases where the BBB remains largely intact (and extravascular contrast uptake is therefore slow) is unclear. Some groups have applied semi-quantitative approaches, comparing signal enhancement–time curves between patient groups to avoid assumptions inherent in modelling (Wardlaw et al., 2008) or calculating heuristic quantities such as “area under curve” (Topakian et al., 2010). These approaches have the advantage of being straightforward to implement and do not require advanced image processing, but they do not distinguish between intra- and extravascular contrast and may be influenced by the acquisition parameters and other variables. Recently, a number of more complex, model-based approaches to quantifying subtle BBB leakage have been suggested, with applications in cognitive impairment (Montagne et al., 2015, Taheri et al., 2011a), healthy subjects (Cramer and Larsson, 2014), and in the normal-appearing tissue of patients with multiple sclerosis (Cramer et al., 2014), primary brain tumours (Larsson et al., 2009, Sourbron et al., 2009) and acute stroke (Thornhill et al., 2010), involving a range of acquisition and analysis methods.

In this work, we performed DCE-MRI in a large cohort of mild stroke patients with a range of small vessel disease features and severity. We aimed to determine the tracer kinetic modelling approach most suitable for assessing subtle BBB leakage using a data-driven approach, to determine its validity both in theory and experimentally and to obtain tracer kinetic parameters for normal-appearing tissues and lesions. We hypothesised that low temporal resolution data analysed using the Patlak model is appropriate for measuring low-level BBB permeability with high spatial resolution and brain coverage, and that scanner instability causes significant systematic errors in quantitative permeability measurements.

## Materials and methods

### Clinical cohort

#### Patients

We recruited 264 patients with first clinically evident mild (i.e., expected to be non-disabling) ischaemic stroke from the local stroke service. Included patients had to be over 18 years old, have a definite diagnosis of ischaemic stroke, be able to consent themselves, have an MRI scan at diagnosis and be medically stable enough to return for a DCE-MRI scan at between 1 and 3 months post-stroke and a follow-up at 1 year. All patients underwent clinical assessment by a stroke physician, diagnostic MR imaging and cognitive testing at presentation. An expert panel of stroke physicians and neuro-radiologists assessed each case in order to confirm the diagnosis of ischaemic stroke and classify the ischaemic stroke subtype. DCE-MRI was performed a minimum of 1 month after the stroke in order to avoid acute effects of the stroke on the local BBB. This study was approved by the Lothian Ethics of Medical Research Committee (REC 09/81101/54) and the NHS Lothian R + D Office (2009/W/NEU/14), and all patients gave written informed consent.

#### MRI

All imaging was performed on a 1.5 T MRI scanner (Signa HDxt, General Electric, Milwaukee, WI) using an 8-channel phased-array head coil. Diagnostic MR imaging at presentation included axial *T*_2_-weighted (T2W; *T*_R_/*T*_E_ = 6000/90 ms, 24 × 24 cm field of view (FoV), 384 × 384 Propeller acquisition, 1.5 averages, 28 × 5 mm slices, 1 mm slice gap), axial fluid-attenuated inversion recovery (FLAIR; *T*_R_/*T*_E_/*T*_I_ = 9000/153/2200 ms, 24 × 24 cm FoV, 384 × 224 acquisition matrix, 28 × 5 mm slices, 1 mm slice gap), gradient echo (GRE; *T*_R_/*T*_E_ = 800/15 ms, 20° flip angle, 24 × 18 cm FoV, 384 × 168 acquisition matrix, 2 averages, 28 × 5 mm slices, 1 mm slice gap) and sagittal 3D *T*_1_-weighted imaging (T1W; inversion recovery-prepared spoiled gradient echo *T*_R_/*T*_E_/*T*_I_ = 7.3/2.9/500 ms, 8° flip angle, 330 × 214.5 cm FoV, 256 × 146 acquisition matrix, 100 × 1.8 mm slices) and diffusion tensor MRI (single-shot echo-planar imaging with 30 diffusion directions (*b* = 1000 s/mm) and 2 × *b*_0_ acquisitions, *T*_R_/*T*_E_ = 7700/82 ms, 24 × 24 cm FoV, 128 × 128 acquisition matrix, 28 × 5 mm slices, 1 mm slice gap). DCE-MRI was performed at approximately 1 month after first presentation and consisted of a 3D T1W spoiled gradient echo sequence with *T*_R_/*T*_E_ = 8.24/3.1 ms, 24 × 24 cm FOV, 256 × 192 acquisition matrix and 42 × 4 mm slices. Two pre-contrast acquisitions were carried out at flip angles of 2° and 12° to enable the calculation of pre-contrast longitudinal relaxation times (*T*_10_). An intravenous bolus injection of 0.1 mmol/kg of gadoterate meglumine (Gd-DOTA, Dotarem, Guerbet, France) was administered simultaneously with the start of 20 acquisitions with 12° flip angle and a temporal resolution of 73 s, leading to a DCE-MRI duration of approximately 24 minutes.

#### Image processing

**Fig. 1:**
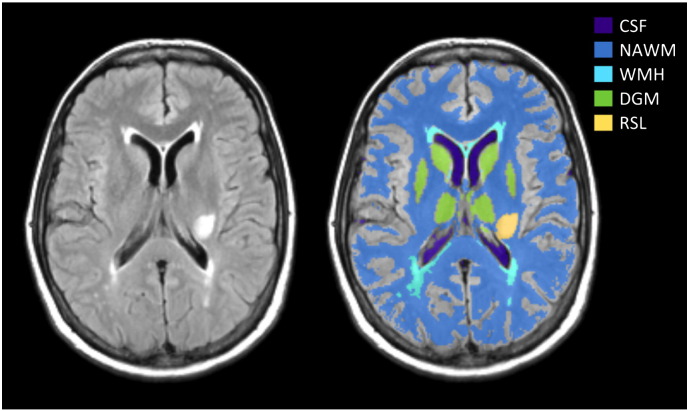
Representative MRI data and tissue segmentation. FLAIR image (left) and tissue masks superimposed on FLAIR image (CSF: cerebrospinal fluid, NAWM: normal-appearing white matter, WMH: white matter hyperintensities, DGM: deep grey matter, RSL: recent stroke lesion).

All image analysis was performed blind to clinical and permeability data. All structural and DCE-MRI images were aligned to the 12° pre-contrast image using rigid-body registration (FSL-FLIRT (Jenkinson and Smith, 2001)) in order to correct for bulk patient movement. We defined all small vessel features according to agreed STRIVE standards (Wardlaw et al., 2013b). We used a multispectral MRI data fusion and minimum variance quantisation technique (Valdés Hernández et al., 2010) for the segmentation of white matter hyperintensities (WMH) and normal-appearing white matter (NAWM). Please note that we use the term “WMH” to include hyperintensities in the white and subcortical grey matter. The resulting masks were manually refined and, separately, old stroke lesions and the index (i.e., which had led to patient entry to the study) stroke lesion boundaries were semi-automatically outlined on FLAIR images using the “Region of Interest” tool of Analyze 11.0^TM^ (AnalyzeDirect, KS). Index/recent stroke lesions (RSL) were defined as the hyperintense regions identified on the diffusion weighted image including any corresponding signal changes on FLAIR, T2W and T1W images, associated with swelling or lack of ex vacuo effect, that followed a vascular territory. Stroke lesion masks were checked for accuracy by a neuroradiologist; all other tissue masks were checked visually for accuracy and manually edited as necessary. Subcortical/deep grey matter (DGM) masks were generated automatically by a software pipeline that used FSL-SUSAN (Smith and Brady, 1997) for noise reduction, an age-relevant brain template (Farrell et al., 2009), FSL-FLIRT for aligning the template to each image data set and FSL-FIRST (Patenaude et al., 2011) for extracting the subcortical structures, followed by manual boundary correction. To minimise any residual contamination of the DGM, the mask was eroded by one voxel. An example of a FLAIR image and segmentation masks is shown in Fig. 1.

#### DCE-MRI analysis

For each post-contrast time point *i*, we calculated the median signal intensity over all voxels for each tissue type (*S_i_*). The signal enhancement *E_i_* (representing the fractional signal increase above baseline) was then calculated as *E_i_* = (*S_i_* − *S*_0_) / *S*_0_, where *S*_0_ is the signal intensity of the 12° pre-contrast acquisition. *T*_10_ was calculated based on the variable flip angle method by Brookes et al. (Brookes et al., 1999) using

(1) $$\frac{1}{T_{10}}=\frac{1}{T_{R}}\ln\left(\frac{S_{R}\sin\alpha_{b}\cos\alpha_{a}-\sin\alpha_{a}\cos\alpha_{b}}{S_{R}\sin\alpha_{b}-\sin\alpha_{a}}\right)\text{,}$$

where *S*_R_ = *S*_a_/*S*_b_, with *S*_a_ and *S*_b_ representing the signal intensities of the two pre-contrast acquisitions with flip angles α_a_ = 2° and α_b_ = 12°. The contrast agent concentration *C_i_* was then estimated by numerical solution of the following equation (Armitage et al., 2005):

(2) $$E_{i}=\exp\left(-r_{2}C_{i}T_{E}\right)\times \left[\frac{1-\exp\left(-P-Q\right)-\cos\alpha_{b}\left(\exp\left(P\right)-\exp\left(-2P-Q\right)\right)}{1-\exp\left(P\right)-\cos\alpha_{b}\left(\exp\left(P-Q\right)-\exp\left(-2P-Q\right)\right)}\right]-1\text{,}$$

where *P =* *T*_R_/*T*_10_, *Q = r*_1_*C_i_T*_R_, longitudinal and transverse contrast agent relaxivities *r*_1_ and *r*_2_ with *r*_1_/*r*_2_ = 4.2/6.7 s^− 1^mM^− 1^ (Rohrer et al., 2005).

We obtained a vascular input function (VIF) from a voxel located on the superior sagittal sinus (SS) since partial volume effects and inflow artefact were reduced at that location compared with obtaining the AIF from a feeding artery (Lavini and Verhoeff, 2010); the delay between arterial and venous responses is expected to be very small compared with the temporal resolution (Sourbron et al., 2009, Sourbron and Buckley, 2012). Two observers, independently and blind to each other's results, manually selected a single voxel for VIF extraction, using a slice proximal to the basal ganglia structures and the lateral ventricles. This voxel was chosen to provide a high peak signal enhancement and smooth variation during the DCE-MRI time course. Where the observers selected different voxels, the voxel with the highest peak enhancement was chosen unless the signal curve was significantly noisier (a noise estimate was calculated as the sum of squared differences between the signal curve and a fitted bi-exponential curve). The calculated whole-blood concentration *C*_b_(*t)* measured in the SS was converted to plasma concentration using the formula *C*_p_(*t*) = *C*_b_(*t*) / (1 *−* Hct), with the most recent available haematocrit measurement in the patient's clinical record (where no haematocrit measurement was available (*n* = 3) we assumed Hct = 0.45).

#### Tracer kinetic modelling

**Fig. 2:**
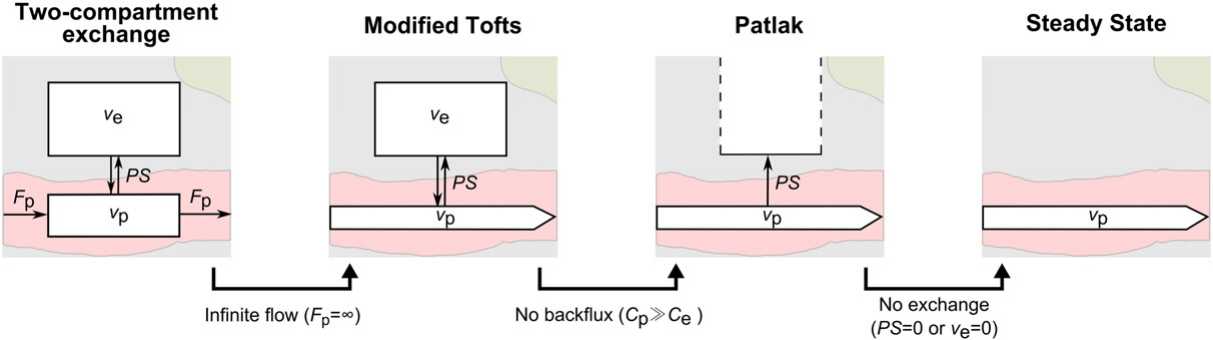
Set of nested tracer kinetic models. Target parameters of DCE-MRI modelling are the fractional plasma volume *v*_p_, the fractional interstitial volume *v*_e_, the plasma flow *F*_p_ and the permeability-surface area product *PS.* The four models are related by a series of simplifying assumptions.

Tracer kinetic modelling aims to provide a link between the tissue signal enhancement and the physiological parameters, including the fractional plasma volume *v*_p_, the fractional interstitial volume *v*_e_ and the permeability-surface area product *PS*. We fitted the following three models to the tissue concentration curves: (i) the modified Tofts model (Tofts et al., 1999), (ii) the Patlak model (Patlak et al., 1983) and (iii) the steady-state model (Sourbron and Buckley, 2013). A schematic overview of these models and their relationship with the more general two-compartment-exchange model (2CXM) (Sourbron and Buckley, 2013) is shown in Fig. 2.

The modified Tofts model describes a highly perfused (*F*_p_* = ∞*) two-compartment tissue, considering bidirectional transport between the blood plasma and the extracellular extravascular space (EES). The concentration of contrast agent in the tissue is given by

(3) $$C_{t}\left(t\right)=v_{p}C_{p}\left(t\right)+K^{\mathrm{Trans}}\int_{0}^{t}C_{p}\left(\tau \right)\;\exp\left[-\frac{K^{\mathrm{Trans}}\left(t-\tau \right)}{v_{e}}\right]d\tau \text{,}$$

where the volume transfer constant *K*^Trans^ represents the rate at which contrast agent is delivered to the EES per volume of tissue and contrast agent concentration of the VIF (*PS* denotes the same transfer constant with respect to capillary plasma concentration); in general, the measured *K*^Trans^ depends on both the plasma flow *F*_p_ and the permeability-surface area product *PS*; however, if the assumptions of the model are met (i.e., flow is high enough and the rate of contrast extravasation is low enough to ensure equal concentration in the arteries and capillary bed) and the measurement process is accurate, then *K*^Trans^ *≈* *PS* is a good approximation (Sourbron and Buckley, 2013). In the work that follows, we use the symbol *K*^Trans^ to indicate experimental or simulated measurements and *PS* to indicate the permeability-surface area product used to generate simulated data.

The Patlak model can be seen as a special case of the modified Tofts model, which ignores backflux from the EES into the blood plasma compartment. Consequently, it only allows for the estimation of the two parameters *K*^Trans^ and *v*_p_:

(4) $$C_{t}(t)=v_{p}C_{p}\left(t\right)+K^{\mathrm{Trans}}\int_{0}^{t}C_{p}\left(\tau \right)\;d\tau \text{.}$$

If it is further assumed that there is no transfer of contrast to the EES, the one-parameter steady-state model (Sourbron and Buckley, 2013) is obtained:

(5) $$C_{t}(t)=v_{p}C_{p}\left(t\right).$$

Model fitting was performed using in-house software programmed in MATLAB (MathWorks, Natick, MA, USA) by non-linear minimisation of the sum of squared residuals. All parameters were restricted to positive values, and *v*_p_ + *v*_e_ was constrained to be less than or equal to 1; fitting was repeated 25 times with different initial values to reduce the probability of selecting local minima (Ahearn et al., 2005). The first three post-contrast time points of all contrast agent concentration curves were omitted from sum of squares calculation since the rapid concentration changes during and immediately after the first pass are not adequately resolved by the acquisition protocol and are not accurately modelled by any of the three nested models (Larsson et al., 2009), resulting in biased parameter estimates that depend on *F*_p_; this was confirmed by computer simulations.

#### Model comparison

The competing models were ranked according to the Akaike information criterion (AIC) (Akaike, 1974), which has been applied in DCE-MRI by several groups (Brix et al., 2009, Ingrisch et al., 2010). The AIC accounts for the trade-off between goodness-of-fit and model complexity, by combining the sum of squared errors *SS_m_* with the number of free parameters *K_m_* associated with the model *m*:

(6) $$\mathrm{AI}C_{m}=N\;\ln\left(\frac{SS_{m}}{N}\right)+2\left(K_{m}+1\right)\text{,}$$

where *N* denotes the number of data points. For small samples, i.e., *N*/*K_m_* < 40 as in our case, the AIC should be extended with a second-order bias correction term (Burnham and Anderson, 2004), giving the small-sample version

(7) $$AICc_{m}=\mathrm{AI}C_{m}+\;\frac{2K_{m}\left(K_{m}+1\right)}{N-K_{m}-1}\text{.}$$

The Akaike weight AW*_m_* describes the probability that the model *m* is best amongst a set of *M* models, following the equation (Luypaert et al., 2012)

(8) $$AW_{m}=\frac{\exp\left(-\Delta_{m}/2\right)}{\sum_{i=1}^{M}\exp\left(-\Delta_{i}/2\right)}\;\mathrm{with}\;\Delta_{m}=AICc_{m}-\min\left(AICc_{1},\ldots \;,\;AICc_{M}\right)\text{.}$$

For a statistical comparison between tracer kinetic parameters, Wilcoxon's signed rank test (since the data were in general not normally distributed) was used.

### Healthy volunteers

To assess signal changes unrelated to the contrast agent, we recruited 15 healthy volunteers that underwent the same DCE-MRI protocol as applied in the clinical cohort but without administration of contrast agent. This study was approved by ACCORD Healthy Volunteer Research Ethics Committee (REC 14/HV/0001), and all volunteers gave written informed consent. We manually placed regions of interest (ROIs) in NAWM and DGM and calculated median signal intensity and enhancement curves for each ROI as described previously. Signal drift was calculated as the overall change in signal intensity per minute and is given as percentage of the time-averaged signal.

In addition to the dynamic sequence, we accurately measured *T*_1_ using the inversion recovery method (Larsson et al., 1988) (IR) in *n* = 7 volunteers. For this purpose, we acquired 2D inversion recovery spin echo echo-planar imaging with multiple inversion times (IR-SE-EPI; *T*_R_/*T*_E_ = 10000/25.4 ms, 24 × 24 cm FOV, 256 × 192 acquisition matrix, 1 × 4 mm slice, *T*_I_ = [100, 340, 580, 820, 1060, 1300, 2000, 3000] ms). *T*_1_ was calculated by fitting the following equation to the median ROI signal intensities (SI):

(9) $$SI=\left|A+B\;\exp\left(-\frac{T_{I}}{T_{1}}\right)\right|$$

*T*_1_ was measured immediately before and after the non-contrast DCE-MRI acquisition, yielding the two measurements *T*_1,pre_ (before DCE-MRI) and *T*_1,post_ (after DCE-MRI). All values are given as mean and standard deviation.

### Simulations

Numerical simulations were performed to systematically investigate model validity. First, we generated a high-resolution (∆*t* = 0.1 s) VIF using the function introduced by Parker et al., (2006), which consists of two Gaussians plus an exponential term modulated by a sigmoid function. In order to generate a simulated VIF with realistic first-pass behaviour that also matched our data at longer times post-injection, we introduced an additional exponential term resulting in the following function:

(10) $$C_{p}\left(t\right)=\frac{1}{1-Hct}\left(\sum n=12\frac{A_{n}}{\sigma_{n}\sqrt{2\pi }}\exp\left(-\frac{{\left(t-T_{n}\right)}^{2}}{2\sigma_{n}^{2}}\right)+\frac{\sum n=12\alpha_{n}\exp\left(-\beta_{n}t\right)}{1+\exp\left(-s\left(t-\tau \right)\right)}\right)\text{.}$$

For the Gaussian functions and sigmoid modulation, which describe the first and second pass of the VIF, we used the parameters given by Parker et al. (*A*_*n*_, *σ*_*n*_, *T*_*n*_, *s*, *τ*). The parameters *α_n_* and *β_n_* were obtained by fitting Eq. (10) to the population-average VIF from our patient data (Supplementary Fig. 1). Simulated tissue concentration curves were generated by convolving this VIF with the impulse response function of the 2CXM, which has four free parameters: *F*_p_, *PS*, *v*_p_ and *v*_e_ (Sourbron and Buckley, 2013). *PS* and *v*_p_ values were chosen to represent the range of values obtained in normal-appearing tissue, WMH and stroke lesions; *v*_e_ was chosen to be 0.2 (Syková and Nicholson, 2008) and *F*_p_ values between 10 and 50 ml/100 g/min were selected to represent typical values for NAWM, WMH and DGM (Brickman et al., 2009). Random noise was added to the curves to give a concentration contrast-to-noise ratio (CNR) similar to that observed in NAWM and DGM in our clinical cohort; the CNR was defined as the peak contrast agent concentration divided by a noise estimate, which was calculated as the standard deviation of the difference between the measured concentration curve and the Patlak model fit (CNR = max[*C*_t_(*t*)] / std[*C*_t_(*t*) *− C*_Patlak_(*t*)]). Simulations were repeated, adding a signal drift to both the tissue curve and the VIF. The added drift was of similar magnitude to that found in the healthy volunteer cohort (values given in the results section). We then down-sampled the VIF and tissue concentration curves to the experimental temporal resolution (∆*t* = 73 s), and these were fitted to the Patlak model as described above. We also calculated the semi-quantitative parameters AUC_norm_ (defined as the area under the tissue enhancement curve divided by the area under the vascular enhancement curve) and the late slope of the enhancement curve (obtained by linear regression of the tissue signal enhancement data). As for the clinical data, the first three post-contrast data points were neglected for calculating the sum of squared residuals during model fitting and for calculation of semi-quantitative parameters. The simulations were repeated 1000 times for every set of parameters to quantify the influence of noise.

## Results

### Patients

**Fig. 3:**
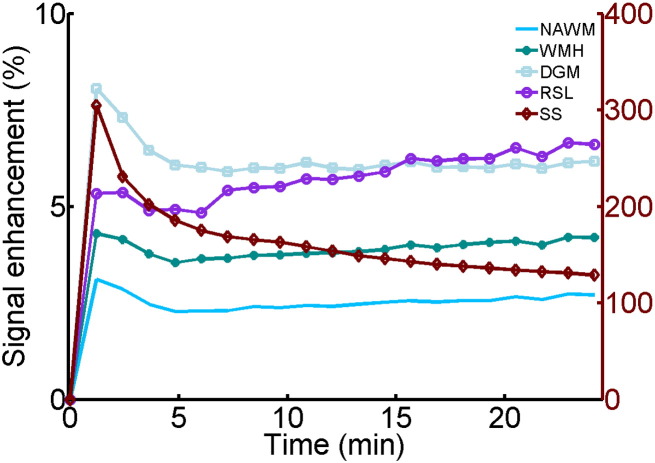
Cohort average signal enhancement curves. Post-contrast signal enhancement versus time obtained from the median signal intensity in each tissue type (NAWM: normal-appearing white matter, WMH: white matter hyperintensities, DGM: deep grey matter, RSL: recent stroke lesion, SS: sagittal sinus) and averaged over all patients (*n* = 201). *Y*-axis scales for the tissue and sagittal sinus enhancement curves are shown on the left and right, respectively.

DCE-MRI was performed on average 38 days after first presentation and data suitable for analysis were obtained in 201 patients with mean age of 66.0 ± 11.5 years. Fig. 3 shows the cohort-averaged signal enhancement curves in NAWM, DGM, WMH, RSL and SS. All tissues show a signal enhancement of approximately 2%–8%, with abnormal tissues (WMH and RSL) showing a steeper increase in signal enhancement over time compared with normal-appearing tissues (WM and DGM).

**Fig. 4:**
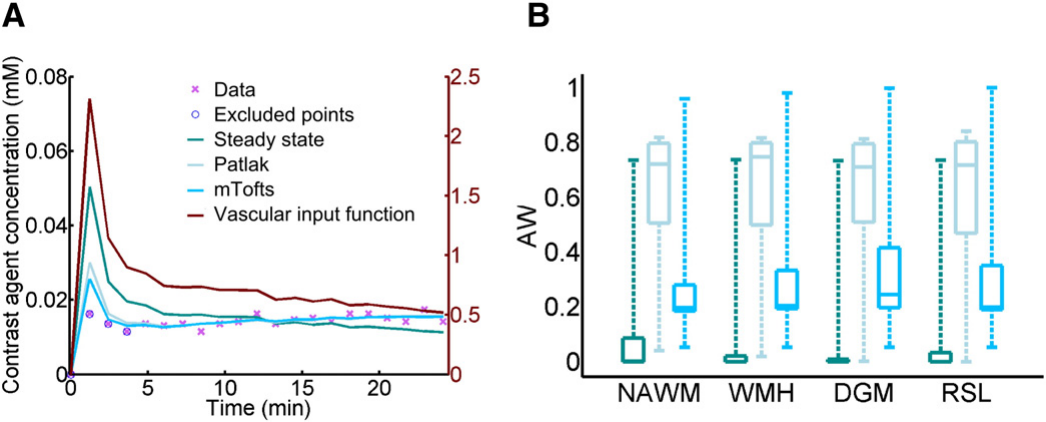
Comparison of model fits to the patient data. (A) Example concentration–time curve for normal-appearing white matter in a single patient. In general, the steady-state model does not fit the data well; while both the Patlak and modified Tofts (mTofts) models typically fit the data similarly well, the Patlak model has a higher Akaike weight (AW) than the modified Tofts model in most cases due to the lower number of free parameters. (B) Comparison of AW for the three models in normal-appearing white matter (NAWM), white matter hyperintensities (WMH), deep grey matter (DGM) and recent stroke lesions (RSL). In most patients, the Patlak model had the highest AW for all tissue types (legend as in A).

**Fig. 5:**
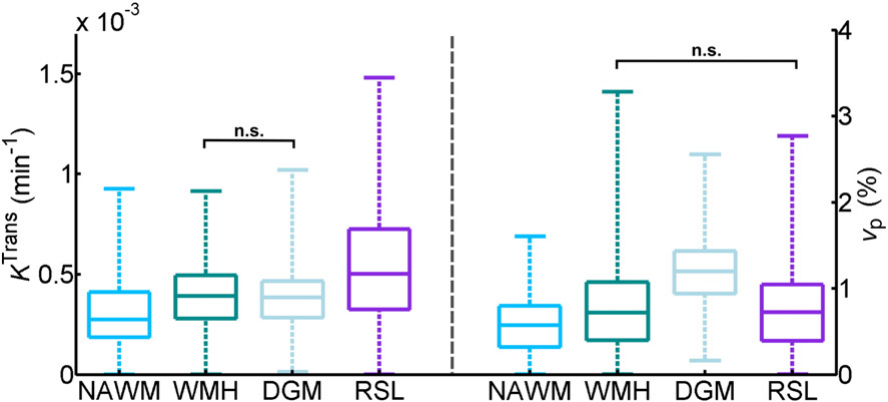
Comparison of fitted Patlak parameters between tissue types. Box plots showing the distribution of *K*^Trans^ (left) and *v*_p_ (right) in normal-appearing white matter (NAWM), white matter hyperintensities (WMH), deep grey matter (DGM) and recent stroke lesions (RSL). Brackets with n.s. indicate non-significant differences with *p* > 0.5; all other differences between tissue types are significant with *p* < 0.001 (brackets omitted for clarity).

An example of tracer kinetic model fitting for a single patient is shown in Fig. 4A; the Patlak and modified Tofts models provide good fits to the measured signal but the steady-state model is not sufficient to describe the data. This observation is confirmed by analysis of the Akaike weights (Fig. 4B), which shows that the Patlak model best represents the data in most subjects for all tissue types, and was selected as the optimum model in 74%–78% of patients for the four tissues measured. The resulting Patlak parameters are listed in Table 1. A comparison between tissue types is shown in Fig. 5; all differences between tissue types were significant (*p* < 0.001) except for *K*^Trans^ in WMH and DGM and *v*_p_ in WMH and RSL. In particular, *K*^Trans^ and *v*_p_ are significantly higher in WMH compared to NAWM; *K*^Trans^ is significantly higher in RSL compared to all other tissues.

### Healthy volunteers

**Fig. 6:**
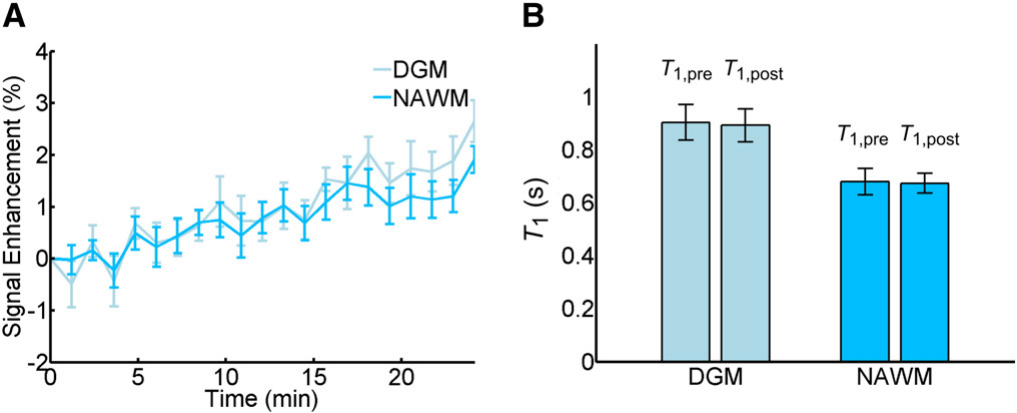
Contrast-free measurements in healthy volunteers. (A) Average signal enhancement curves (*n* = 15) in normal-appearing white matter (NAWM) and deep grey matter (DGM), showing a drift in signal intensity; error bars indicate the mean ± standard error. (B) *T*_1_ measurements obtained before and after the DCE-MRI sequence using the inversion recovery method (*n* = 7); error bars indicate the mean ± 1.96 standard deviations.

DCE-MRI data without contrast administration was obtained in 15 healthy volunteers with mean age 31.4 ± 7.4 years. Fig. 6A shows the corresponding average signal enhancement curves in NAWM and DGM. The data show an approximately linear drift in signal intensity of 0.10 ± 0.06%/min in DGM and 0.06 ± 0.03%/min in NAWM. To investigate whether part of the drift can be explained by subtle physiological changes, we accurately measured *T*_1_ before and after the DCE-MRI sequence in 8 of the volunteers. *T*_1_ values were in line with previous literature (Vymazal et al., 1999) with *T*_1,pre_/*T*_1,post_ = 903 ± 66 ms / 892 ± 53 ms in DGM and *T*_1,pre_/*T*_1,post_ = 680 ± 40 ms / 674 ± 41 ms in NAWM (see Fig. 6B); the small decrease in *T*_1_ over the period of the DCE-MRI acquisition was not statistically significant (Δ*T*_1_ = − 11 ± 20 ms in DGM and Δ*T*_1_= − 7 ± 14 ms in NAWM).

### Simulations

**Fig. 7:**
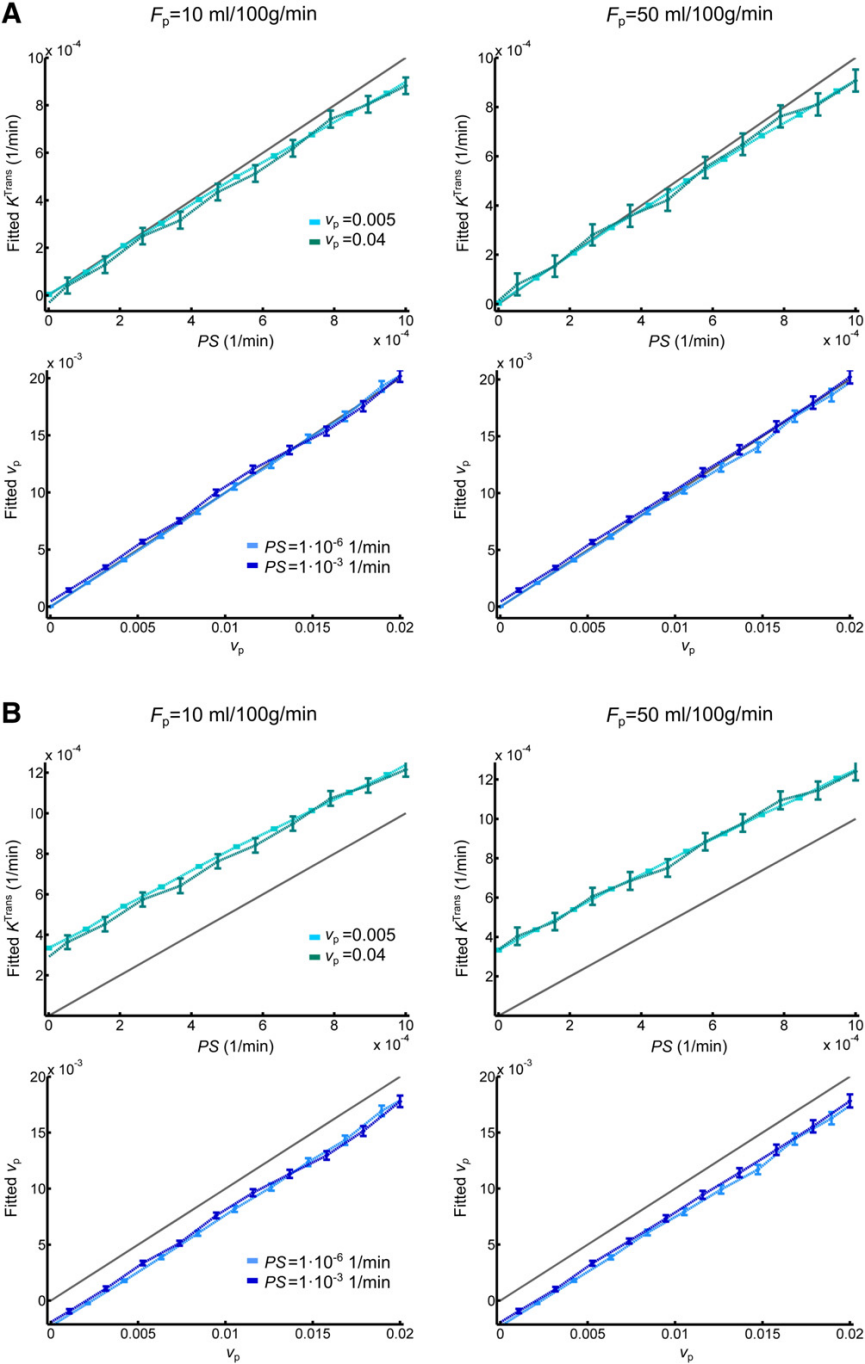
Simulated accuracy of Patlak parameters. (A) Relationship of permeability-surface area product *PS* (top row) and blood plasma volume *v*_p_ (bottom row) values, with corresponding fitted Patlak parameters. Results are shown for two different blood plasma flow (*F*_p_), *PS* and *v*_p_ values. For all simulations, the interstitial volume was set to 0.2. Error bars indicate the mean ± 1.96 standard deviations; the grey line represents the identity line. (B) As above but including a 0.08%/min signal drift.

The results of simulations performed to determine the validity of the Patlak model are shown in Fig. 7. These compare fitted parameters with the “true” parameters used to generate synthetic signal-time curves using the 2CXM model. All simulations are based on a CNR of 8 and a *T*_1_ of 969 ms, corresponding to typical NAWM values observed in our patient cohort. In the absence of drift (Fig. 6A), *K*^Trans^ *≈* *PS* and *v*_p_ is accurately estimated when *PS* is low, despite very low temporal resolution and the simplifying assumptions of the Patlak model. At higher *PS* values, *K*^Trans^ underestimates *PS*, and *v*_p_ is slightly overestimated as a result of back-diffusion, which is neglected in the Patlak model. The model is robust to differences in blood flow, with low flow resulting in a slightly greater underestimation of *PS*. To investigate the effect of scanner drift, we included a 0.08%/min signal drift (comparable to that measured in the healthy volunteer group) in the simulations. This leads to systematic underestimation of *v*_p_ and overestimation of leakage (Fig. 6B), but *K*^Trans^ and *v*_p_ remain approximately linear functions of the specified values, largely independent of plasma flow and of one another. The magnitude of the systematic error introduced by signal drift is *T*_1_ dependent, with an estimated range across the tissues of approximately 2.2–3.2 × 10^− 4^ min^− 1^ for *K*^Trans^ and − 2.4 to − 1.6 × 10^− 3^ for *v*_p_.

**Fig. 8:**
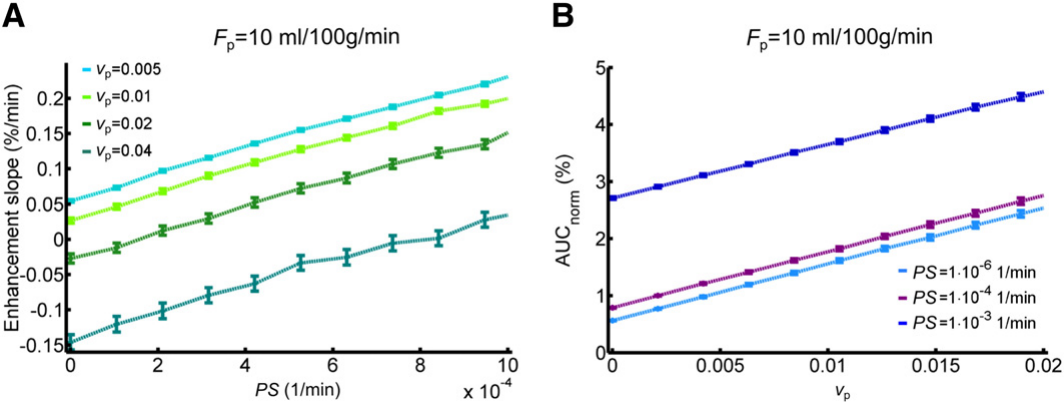
Relationship between simulated semi-quantitative parameters and tissue properties. (A) Relationship between the signal enhancement slope and the permeability-surface area product *PS* for different blood plasma volumes *v*_p_. (B) Relationship between normalised area under the signal enhancement curve and *v*_p_ for different *PS* values. A signal drift of 0.08%/min was added to the synthetic data; results are shown for blood plasma flow *F*_p_ = 10 ml/100 g/min and interstitial volume *v*_e_ = 0.2.

For comparison, Fig. 8 shows the relationship between *PS* and *v*_p_, and the semi-quantitative parameters AUC_norm_ and the late slope of the signal enhancement curve. AUC_norm_ correlates strongly with *v*_p_ but is also influenced by *PS*. Similarly, the enhancement slope correlates with *PS* but is not independent of *v*_p_.

## Discussion

Analysis of patient DCE-MRI data using the Akaike information criterion revealed the Patlak model to be the most appropriate of the three models for quantification of subtle BBB disruption, in line with our hypothesis. The steady-state model does not adequately fit the data; while both the Patlak and modified Tofts models fit the data similarly well, the simpler Patlak model does so using fewer free parameters and is therefore selected. The additional complexity of the modified Tofts model results in over-fitting in the low-permeability regime, consistent with a previous report regarding the behaviour of Tofts parameters in a smaller study of healthy volunteers and MS patients (Cramer and Larsson, 2014). Application of the Patlak model in situations of subtle BBB leakage was further supported by the numerical simulations, confirming that appropriate estimates of *PS* (*≈ K*^Trans^), and *v*_p_ can be obtained independent of cerebral blood flow (CBF) and despite the use of a temporal resolution much lower than is typically employed in DCE-MRI (Heye et al., 2014); this was consistent with simulations performed by Larsson et al. for a shorter, high temporal resolution acquisition (Larsson et al., 2009). A consequence of this finding is that DCE-MRI protocols for investigating diffuse subtle BBB pathology may benefit from prioritising, as here, spatial resolution, whole-brain coverage and contrast-to-noise ratio over sampling rate to allow for analysis of subtle leakage across all brain structures. In contrast to the fitted tracer kinetic parameters, we found semi-quantitative parameters to reflect a combination of underlying tissue properties.

In our cohort of mild stroke patients, *K*^Trans^ was greater in WMH than in NAWM. This is consistent with some pathology studies (e.g., Tomimoto et al., 1996, Wharton et al., 2015, Young et al., 2008) and other DCE-MRI studies that reported increased BBB disruption in areas of WMH compared to NAWM (Hanyu et al., 2002, Taheri et al., 2011a, Topakian et al., 2010). In contrast, a recent study by Huisa et al. found increased BBB disruption in NAWM surrounding the WMH rather than in the WMH themselves (Huisa et al., 2015), while others failed to detect any BBB leakage in or around WMH (Wahlund and Bronge, 2000). The exact role of BBB breakdown in SVD pathology remains to be defined. *K*^Trans^ was greater still in the recent stroke lesion, comparable to findings of Thornhill et al. in acute-phase stroke lesions (Thornhill et al., 2010) and known tissue changes from many pathology examinations. *K*^Trans^ was greater in GM than in NAWM, which may partly reflect the higher vessel density and vascular surface area of GM (Schlageter et al., 1999), consistent with the higher measured blood plasma volume. WMH, which are often regarded as “ischaemic” lesions and might therefore be expected to have reduced plasma volume, had greater *v*_p_ than NAWM. This finding is only partly explained by the *T*_1_ difference between the tissues, which results in a predicted greater underestimation of *v*_p_ in NAWM due to scanner drift. Assumptions such as tissue-independent relaxivity and water exchange rates may also influence the values. Another possible explanation is that a few WMH are found in tissue that was originally subcortical grey matter, especially in the head of the caudate nucleus, where baseline blood volume is likely to be higher. Very few studies have measured cerebral blood volume (CBV) and CBF in WMH themselves, as opposed to general CBV and CBF in patients with WMH. While some studies using DSC-MRI have reported reduced CBV in WMH, e.g., Sachdev et al. (2004), Marstrand et al. (2002) found reduced CBF but similar CBV in WMH compared with NAWM. Although DCE-MRI is a more quantitative marker of microvascular properties than DSC-MRI, previous studies applying DCE-MRI in SVD have not reported *v*_p_ values for WMH to the best of our knowledge. We suggest therefore that future studies should assess CBF and CBV in WMH as well as normal tissues so as to resolve this issue.

In subjects who did not receive contrast, we observed a signal change of approximately 0.08%/min. This level of instability is unlikely to be problematic in typical DCE-MRI applications with larger, more rapid signal enhancements over shorter acquisition times. However, our simulations show that the drift is predicted to cause a substantial upward shift of *K*^Trans^ measurements in the low-permeability regime, which would account for some of the apparent leakage seen in normal-appearing white matter and other tissues. As a result, attempts to quantify low levels of permeability should be interpreted with caution, unless information regarding scanner drift and its likely effect on the values are available. This finding is consistent with recently reported simulation results (Barnes et al., 2015) and may partly account for the wide variation in *K*^Trans^ values reported in the literature for normal-appearing brain tissue (Cramer et al., 2014, Larsson et al., 2009, Montagne et al., 2015, Sourbron et al., 2009, Taheri et al., 2011a, Taheri et al., 2011b, Thornhill et al., 2010), which span three orders of magnitude and include negative values (Sourbron et al., 2009). Signal drift measurements are rarely reported in the DCE-MRI literature, an exception being Cramer et al. who reported a drift at 3 T (1%–3% over 15 minutes) comparable to our measurements at 1.5 T (Cramer and Larsson, 2014). The drift behaviour of our scanner was also within the range of values obtained in a multi-centre survey of fMRI quality assurance parameters, which concluded that “stable scanners generally average around 1.0% [signal drift over 6.7 minutes] or less” (Friedman and Glover, 2006). The influence of signal drift in fMRI is much less severe than here since it typically has a lower frequency than the task-related signal change, is suppressed using a high-pass filter and the scans are quicker; in DCE-MRI, it is harder to distinguish between drift and contrast enhancement as both occur on similar timescales. It is worth noting that DCE-MRI methods consisting of repeated *T*_1_ measurements (e.g., Taheri et al., 2011b) should largely self-compensate for signal drifts of instrumental origin and, depending on the pulse sequence used, changes in the radiofrequency field strength. However, the causes of signal drift are not well-understood and may also be influenced by subtle biological changes (Lowe and Russell, 1999), consistent with our (non-significant) observation of *T*_1_ change and by a previous report of tissue-dependent drift on the same scanner as in the present work (Armitage et al., 2011). The effect should also be mitigated by optimising the protocol for sensitivity to changes in contrast agent concentration.

Despite the potentially confounding influence of drift, our simulations indicate that *K*^Trans^ and *v*_p_ estimates remain approximately linear independent functions of the permeability-surface area product and plasma volume, respectively. They provide a valuable if relative indication of blood–brain barrier integrity and blood plasma volume for applications in well-designed studies with appropriate control groups and statistical correction for confounds; such quantities remain easier to interpret than semi-quantitative measurements (Budde et al., 2012).

This and other DCE-MRI studies are limited by additional sources of error, which have been described elsewhere. For example, the variable flip angle method (also known as “DESPOT1”) used here and in many other studies (Homer and Beevers, 1985) has the advantage of yielding *T*_1_ maps with whole-brain coverage and adequate SNR within an acceptable acquisition time. However, it is sensitive to flip angle variations across the brain, i.e., deviations of the actual from the operator defined flip angles caused by *B*_1_ field inhomogeneities and the slab excitation profile of the radiofrequency pulse, resulting in errors in *T*_10_ and other parameters (Armitage et al., 2011, Schabel and Parker, 2010). At 1.5 T and using a radiofrequency transmit volume coil, such flip angle variations should be limited and reasonably consistent within the cohort. In future studies, especially on scanners with higher field strength, it would be prudent to either employ methods that estimate and/or correct for flip angle errors, such as DESPOT1-HIFI (Deoni, 2007). Look–Locker-based methods such as TAPIR (Shah et al., 2001) also provide an alternative approach for *T*_10_ measurement, permitting faster sampling of the magnetization recovery curve than the conventional inversion recovery method. The sensitivity of these methods to flip angle variations is reduced since the flip angle can be modelled as an unknown parameter during fitting of the inversion recovery curve. It should also be recognised that all tracer kinetic models necessarily make assumptions regarding tissue structure and the transport of blood plasma and contrast agent. For example, as the low temporal resolution of our acquisition protocol does not permit estimation of blood flow, we selected three models that assume the tissue to be highly perfused (i.e.,* = ∞ F*_p_). Although this is not the case in real tissues, it remains a good approximation when the blood flow is sufficient to equalise the arterial and capillary concentrations (requiring *PS* << *F*_p_) (Sourbron and Buckley, 2011), with our simulations confirming the validity of this approximation; the choice of models should also be seen in relation to temporal resolution, which in this case is much longer than the transit time of the tissue blood compartment. It should also be emphasised that while *PS* (or *K*^Trans^) is commonly used as a marker of “permeability,” it is equally influenced by the capillary surface area, which will depend on the anatomy and pathology of micro vessels. Future studies should try to determine vessel density and size so as to include realistic estimates of capillary endothelial surface area in the permeability calculations. Furthermore, a disadvantage of low temporal resolution acquisition is that cerebral blood flow cannot be determined in addition to *v*_p_ and *PS*; where knowledge of CBF is required and temporal resolution is adequate, the uptake model may be substituted for the Patlak model in low-permeability tissue (Ingrisch et al., 2012, Sourbron et al., 2009). Finally, the DCE-MRI data in this study has been analysed at the level of ROIs rather than voxels. This approach was selected due to the low contrast-to-noise ratio in single voxels and due to the influence of artefacts (e.g., Gibbs ringing and motion), which, while typically at the level of only a few percent, have a similar magnitude to the small contrast-induced signal *changes* and therefore have a disproportionate influence on voxel-wise pharmacokinetic parameters (Supplementary Fig. 2). Averaging over an ROI reduces the influence of noise and artefact, enabling more robust measurement of background BBB status, especially in normal-appearing tissue where signal changes are small. However, a limitation of this approach is that it does not allow the detection of local variation in BBB function.

In conclusion, the Patlak model is a simple and appropriate method for measuring low-level BBB leakage, and our results, based on a large sample of mild stroke patients, justify its emerging popularity in the study of disorders involving subtle BBB disruption and of healthy-appearing tissue (Cramer et al., 2014, Montagne et al., 2015, Taheri et al., 2011a). The model is reasonably robust to the assumptions of high blood flow and negligible back-diffusion, but the resulting tracer kinetic parameters are influenced by signal drift particularly at low-permeability states. It is therefore important to optimise study MRI protocols for measurement of low-level permeability and to assess the magnitude and consistency of drift in future studies by performing non-contrast experiments in volunteers as done here or in a subset of patients (Armitage et al., 2011, Cramer and Larsson, 2014) and simulations to predict the likely effect on study findings. These considerations are particularly critical for multi-centre studies.

The following are the supplementary data related to this article.

Supplementary Fig. 1 High temporal resolution VIF generated for the simulation study. The VIF was generated based on the generic function introduced by Parker et al., yielding realistic first-pass behaviour. In order to match our clinical data measured at longer times post-injection, the function was extended by an additional exponential term.

Supplementary Fig. 2 Example voxel-by-voxel maps of Patlak parameters. FLAIR images, spoiled gradient echo images (SPGR), *K*^Trans^ (min^− 1^) maps and *v*_p_ maps are shown for two different patients (A and B). Both patients exhibit a recent stroke lesion, which is visible in the FLAIR (indicated by white arrow) and diffusion weighted image. A corresponding area of increased BBB disruption can be seen in the *K*^Trans^ maps. While the pharmacokinetic parameter maps for patient A are acceptable, those of patient B are strongly influenced by low-level motion artefact, even though it is barely visible in the raw SPGR images. Such artefacts restrict the utility of voxel-wise analysis.

## Figures and Tables

**Table 1:** Fitted Patlak parameters. Values are shown as mean ± standard error in normal-appearing white matter (NAWM), white matter hyperintensities (WMH), deep grey matter (DGM) and recent stroke lesions (RSL). All differences between tissue types were significant (*p* < 0.001) except for *K*^Trans^ in WMH and DGM and *v*_p_ in WMH and RSL.

	*K*^Trans^ (× 10^− 4^ min^− 1^)	*v*_p_ (× 10^− 2^)
NAWM	2.96 ± 0.12	0.58 ± 0.02
WMH	3.96 ± 0.13	0.80 ± 0.04
DGM	3.91 ± 0.12	1.21 ± 0.03
RSL	5.77 ± 0.41	0.80 ± 0.05
