# Supplementary figures and images for: Tracer kinetic modelling for DCE-MRI quantification of subtle blood–brain barrier permeability
Source: Neuroimage. 2016 Jan 15;125:446–55. doi: 10.1016/j.neuroimage.2015.10.018 (PMC4692516; doi:10.1016/j.neuroimage.2015.10.018)

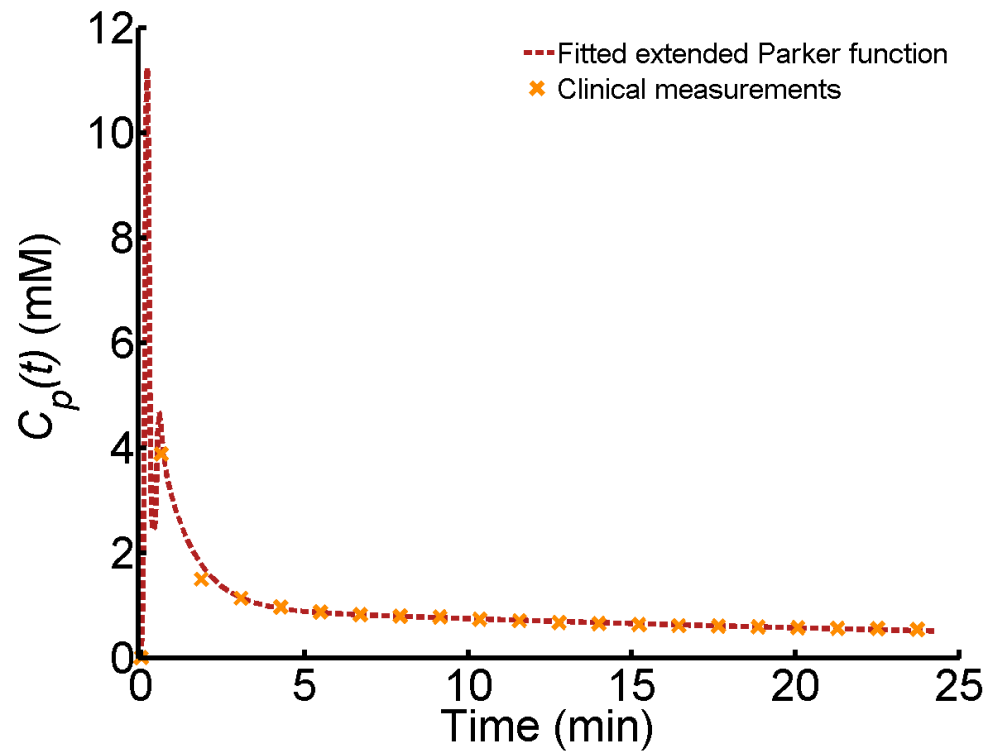

Supplement: Supplementary Fig. 1 — High temporal resolution VIF generated for the simulation study. The VIF was generated based on the generic function introduced by Parker et al., yielding realistic first-pass behaviour. In order to match our clinical data measured at longer times post-injection, the function was extended by an additional exponential term. [file mmc1.pdf]

**A**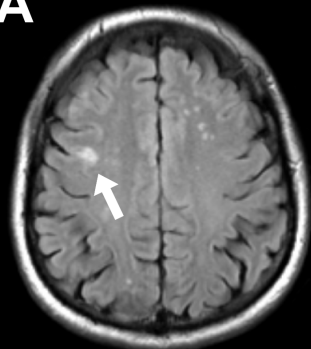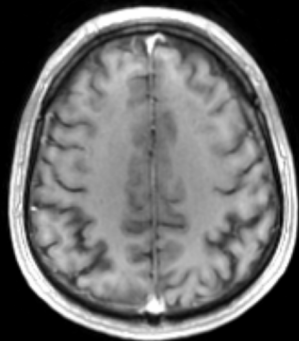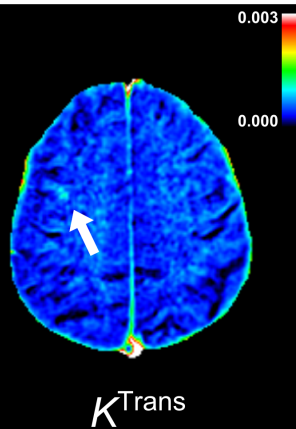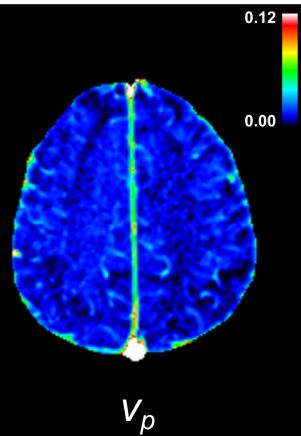**B**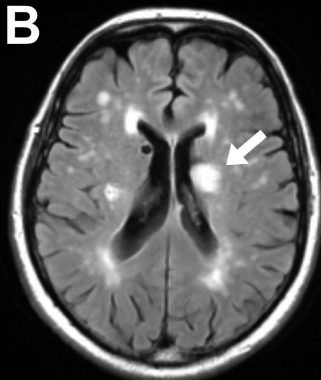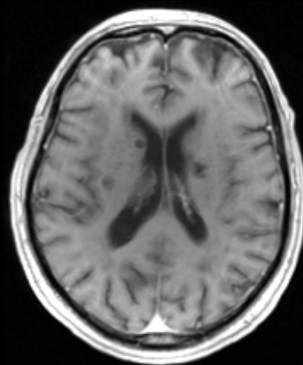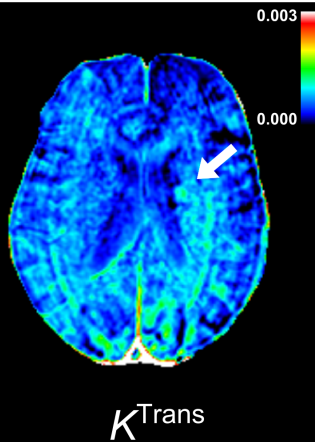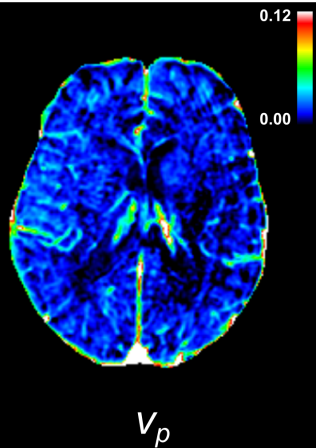

Supplement: Supplementary Fig. 2 — Example voxel-by-voxel maps of Patlak parameters. FLAIR images, spoiled gradient echo images (SPGR), KTrans (min− 1) maps and vp maps are shown for two different patients (A and B). Both patients exhibit a recent stroke lesion, which is visible in the FLAIR (indicated by white arrow) and diffusion weighted image. A corresponding area of increased BBB disruption can be seen in the KTrans maps. While the pharmacokinetic parameter maps for patient A are acceptable, those of patient B are strongly influenced by low-level motion artefact, even though it is barely visible in the raw SPGR images. Such artefacts restrict the utility of voxel-wise analysis. [file mmc2.pdf]
